# Sociobiological Control of Plasmid Copy Number in Bacteria

**DOI:** 10.1371/journal.pone.0009328

**Published:** 2010-02-24

**Authors:** Mukta M. Watve, Neelesh Dahanukar, Milind G. Watve

**Affiliations:** Indian Institute of Science Education and Research, Pune, Maharashtra, India; University of California, United States of America

## Abstract

All genes critical for plasmid replication regulation are located on the plasmid rather than on the host chromosome. It is possible therefore that there can be copy-up “cheater” mutants. In spite of this possibility, low copy number plasmids appear to exist stably in host populations. We examined this paradox using a multilevel selection model. Simulations showed that, a slightly higher copy number mutant could out-compete the wild type. Consequently, another mutant with still higher copy number could invade the first invader. However, the realized benefit of increasing intra-host fitness was saturating whereas that of inter-host fitness was exponential. As a result, above a threshold, intra-host selection was overcompensated by inter-host selection and the low copy number wild type plasmid could back invade a very high copy number plasmid. This led to a rock-paper-scissor (RPS) like situation that allowed the coexistence of plasmids with varied copy numbers. Furthermore, another type of cheater that had lost the genes required for conjugation but could hitchhike on a conjugal plasmid, could further reduce the advantage of copy-up mutants. These sociobiological interactions may compliment molecular mechanisms of replication regulation in stabilizing the copy numbers.

## Introduction

Plasmids are extra chromosomal elements of circular DNA in bacteria, which replicate independent of the host genome. Plasmids exploit the machinery of host cell for their replication, but many of them carry useful or conditionally advantageous genes and therefore cannot be generalized as parasites. Many experiments have shown that the host cell has to bear a cost for carrying a plasmid [1], [2]. The cost is highly variable and ranges from undetectably small to as large as 40% [3]. The cost can be ameliorated by host parasite coevolution [4]–[6]. Although there are a number of confounding factors, the cost can be generally assumed to increase with copy number and the length of the plasmid [7], [8]. However, plasmids impart a wide range of unique features to the host by contributing to metabolic versatility and resistance to environmental factors [9], [10]. Therefore it has been argued that in the presence of positive selection for a plasmid borne gene the plasmid can be stable. However the presence of useful genes does not seem to be central to the evolution and stability of plasmids because a useful gene could ultimately be incorporated in the bacterial chromosome [11] saving the cost of carrying the plasmid. In the case of genes involved in extracellular products such as virulence factors, a ‘cheater’ strain that does not produce the virulence factor can invade the virulent population. Having such genes on plasmids make horizontal gene transfer possible coercing the cheaters to make the extracellular products [12]. Although such systems can be robust to bacterial cheaters, they may not be robust to plasmid cheaters with the gene for the extracellular products deleted. Such plasmids will have an equal chance of horizontal transfer but the shorter plasmids could replicate faster and replace the wild type. Therefore although many plasmids carry useful genes, that does not appear to be necessary and sufficient cause of plasmid stability.

Furthermore, it has been suggested that in the absence of selection for any plasmid borne gene a plasmid free cell should have a selective advantage and the stability of the plasmid is difficult to explain. Factors such as spread of the plasmid by conjugal transfer to compensate for the loss in host fitness, compensatory mutations, co-evolution of host and plasmid to reduce the cost [4]–[6] or plasmid addiction [13], [14] are some of the possible mechanisms responsible for maintenance of a plasmid.

Many mathematical models have focused on the problem of persistence of plasmid in the host cells [11], [15], [16]. More perplexing is the problem of stability of copy number of a plasmid in the host cell. This is one of the many paradoxes in the evolution and stability of plasmids [13]. Most of the plasmids are considerably smaller than the chromosome in terms of length of DNA. Therefore if the same machinery is being used for replication, there can be many replication cycles of the plasmid per single replication of the chromosome. This can result into rapid escalation of plasmid numbers in a cell leading to increased metabolic burden on the host cell and eventually cell death [17]. This can be prevented only by tight regulation of plasmid replication [13], [18], [19]. Ironically, all critical genes involved in the regulation of plasmid replication are on the plasmid. This raises a potential evolutionary problem. A low copy number is optimal for long term survival of the plasmid in a host cell lineage. However, any mutation that loosens the control and thereby increases the copy number has a short term advantage over the wild type although it may affect long term stability of the system. Therefore copy-up mutations can be considered as “cheaters” and such cheaters can invade the wild type and therefore low copy number is unlikely to be stable. Plasmids with copy numbers as high as 200 are observed in natural populations [20], [21] indicating that the problem is not hypothetical alone. Presence of high copy number plasmids and the stability of low copy number plasmids in spite of the potential threat of invasion is very much a real life paradox.

A number of mechanisms exist for the regulation of plasmid replication and one or more of these mechanisms can control the plasmid replication separately or in combination [9], [22]–[24]. Paulsson [16] recognized the conflict between two levels of selection in the control of plasmid copy numbers. Plasmids that systematically over-replicate relative to their cell mates have a higher chance of fixing in a cell (intra-host selection). However cells having such plasmids have to pay a greater cost and therefore are likely to be competed out by hosts with low copy numbers or ones without a plasmid (inter-host selection). Paulsson [16] suggested that intra-host selection should favor evolution of *cis* acting activators while inter-host selection favors evolution of retaliation by *trans* acting inhibitors of replication. However, such refinements in the mechanisms of replication control and reorganization of genes involved do not rule out the possibility as well as selective advantage of copy-up mutants. The demonstration of copy-up mutants in spite of multiple mechanisms of replication regulation [23] implies that the molecular mechanisms of replication regulation are not infallible and therefore unable to explain the evolutionary stability of copy numbers.

There can be multiple mechanisms by which a copy-up mutant can outcompete a low copy wild type. The effective growth rate of the mutant in terms of the number of replications per host cell cycle is greater than the wild type resulting into progressive dilution of the wild type. Since the wild type replication gets inhibited at a lower copy number and if the copy number control mechanism operates in response to the total copy number, the mutant can suppress the replication of the wild type. Also if we assume that a random copy is transferred in a conjugational event, the probability of getting transferred will be higher for a copy-up mutant. Despite a multiplicity of potential advantages of high copy numbers, plasmids with low copy numbers are surprisingly common. We therefore need to look at other mechanisms for the stability of low copy number plasmids.

It is known that host cells already bearing a plasmid are at least partially immune to conjugal intake of another plasmid [25]. This is a likely mechanism by which horizontal invasion by copy up mutants can be arrested. It has not been critically examined theoretically or experimentally whether this mechanism is necessary and sufficient to prevent high copy number cheaters. Although a host cell lineage having a low copy number plasmid may be immune to conjugational invasion by another plasmid, mutants can nevertheless arise within this population and get transmitted vertically in the cell lineage as well as horizontally to plasmid free cells. In order to test whether this mechanism is necessary to prevent invasion by high copy number mutants, the best approach would be to start with a model without any such mechanism and see whether stability of low copy numbers is possible.

We explore here the possibility that copy numbers are selected by complex sociobiological interactions and are always in a dynamic steady state emerging out of conflicting levels of selection. We use a multilevel selection approach similar to Paulsson [16] but instead of looking at the evolution of molecular mechanisms we focus on the long term population dynamics of different types of plasmid and host populations in a competitive environment.

Apart from copy numbers another type of social cheating is possible in plasmid populations. Plasmids spread by conjugal transfers and the genes for conjugal mechanisms are borne by plasmids. There is likely to be a cost to the plasmid (in terms of increased replication time) in carrying the conjugal genes and making the conjugal machinery would be a cost to the host cell. Non-conjugal plasmids can arise as cheaters in such a system and hitch-hike on conjugal plasmids during conjugal transfer. Using our model system we also examine how the two types of cheaters would interact.

## Methods

### The Model

We assumed four different variants or mutants of a plasmid. The wild type or *lc* is a low copy number plasmid with stringent control over replication and having conjugal abilities (Table 1). Plasmid *ln* is a mutant with deletion of the gene cluster required for conjugation (such as the *tra* gene complex) but retains constraint on copy number. Plasmid *hc* is a mutant with less constrained replication regulation resulting into higher copy number, but retaining the *tra* gene complex. Plasmid *hn* is a mutant of *hc* that has lost the *tra* gene complex. The host cells can be free of plasmid (*x*_0_) or have one of the 4 types of plasmids (*x_lc_*, *x_ln_*, *x_hc_*, *x_hn_* respectively) or can have multiple infections (*x_m_*, see below). The cost of carrying a plasmid which is assumed to be directly proportional to the copy number (*g_lc_*, *g_ln_*, *g_hc_*, *g_hn_*) affects the intrinsic growth rates of the five types of host populations. The cost of carrying the *tra* gene complex is also assumed to affect the rate of replication of the plasmids as well as the host cells carrying them. Therefore the intrinsic growth rate of a host cell type (*r_lc_*, *r_ln_*, *r_hc_*, *r_hn_*) is calculated as the baseline fitness (*r*_0_, assumed to be 1) minus the cost of carrying a particular plasmid and the cost of conjugation if the plasmid bears the conjugal machinery. Therefore,
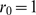


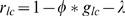


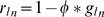


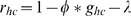


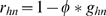
Where *φ* is the cost paid by the host for the intra-host fitness of plasmid, *λ* is the cost of conjugation for the host and *g_i_* is the intra-host fitness of plasmid of type *i* (where *i* ∈ *lc*, *ln*, *hc*, *hn*). Fitness of the given plasmid can be given as
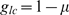


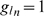


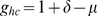


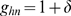
Where *μ* is the cost of carrying conjugal genes and *δ* is the additive fitness due to extra copy number of the plasmid. We assume that the fitness *δ* is a linear function of plasmid copy number. The growth rates *g_i_* reflect the competitive advantages of different types of plasmids.

**Table 1:** Symbols used in the model with description.

Symbol	Description
*lc*	Low copy number plasmid with conjugation ability
*ln*	Low copy number plasmid without conjugation ability
*hc*	High copy number plasmid with conjugation ability
*hn*	High copy number plasmid without conjugation ability
*x*_0_	Frequency of plasmid free cell
*x_i_*	Frequency of cell infected by plasmid type *i* *
*x_m_*	Frequency of cell infected by multiple types of plasmid
*r*_0_	Intrinsic growth rate of plasmid free cell
*r_i_*	Intrinsic growth rate of cell infected by plasmid type *i*
*r_m_*	Intrinsic growth rate of cell infected by multiple types of plasmid
*g_i_*	Fitness of plasmid of type *i*
*p_i_*	Frequency of plasmid of type *i* in host cell with multiple infection
*φ*	Cost paid by the host for the fitness of plasmid
*λ*	Cost of conjugation for the host
*μ*	Cost of conjugal efficiency for the plasmid
*δ*	Additive fitness due to extra copy number of the plasmid
*ε*	Rate of plasmid curing
*β_l_*	Conjugation frequency of the low copy number plasmid (*lc*)
*β_h_*	Conjugation frequency of the high copy number plasmid (*hc*)

*i ∈ *lc*, *ln*, *hc*, *hn*.

In the base line model we do not assume any plasmid incompatibility and multiple infections are permitted. The four types of plasmids could result in many possible combinations of plasmids in cells with multiple infections. However, for simplicity of the model we considered only a single category *x_m_* that represented the pooled population of all cells with multiple infections having different proportions (*p_lc_*, *p_ln_*, *p_hc_*, *p_hn_*) of the four plasmids. Although this assumption is naïve, it can be seen that it is unlikely to affect the model results qualitatively. For example if we make a limiting assumption that all types of plasmids co-occur in a single cell, the probability that a non-conjugal *ln* type of plasmid will get transferred by hitch-hiking on the conjugal plasmid *lc* can be assumed to be a function of the proportion of *lc* and that of *ln*. Thus the transfer rates can be given as *β_l_ p_lc_ p_ln_*. If on the other hand we assume that plasmid types co-occur only in pairs then a non-conjugal plasmid can get hitch hiked only when it pairs with a conjugal plasmid. The probability of the two co-occurring in a host cell will be a function of the proportion of conjugal plasmid and that of non-conjugal plasmid which can be written as *p_ln_ p_lc_* and the rate of plasmid transfer as *β_l_ p_lc_ p_ln_*. Thus both the limiting assumptions give rise to very similar mathematical forms. Therefore we believe that treating multiple infections as a single category is unlikely to bias the results. Treating every possible combination of plasmids in mixed infection will add 11 more population types making the model highly complex and therefore to avoid the complexity we merged them into a single category. The fitness of multiple infected cell (*r_m_*) was calculated by taking the weighted average cost of all plasmids. Plasmid curing was assumed to take place spontaneously with a constant rate *ε*. The assumption that plasmid curing occurred at a constant rate was not completely arbitrary. For plasmids with higher copy number the probability of curing due to stochastic unequal segregation is low by chance alone while for plasmids with low copy number selection favors evolution of efficient partition system reducing the chances of spontaneous curing [16]. As a result, the plasmid curing rate need not scale with copy number.

**Figure 1:**
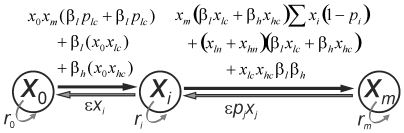
Transformations of cell types due to conjugation and segregation of plasmids. Only one plasmid is assumed to get transferred or get cured at a time. Therefore *x*_0_ cannot be directly transformed into *x_m_* and vice versa. Black arrows indicate conjugation and grey straight arrow indicates curing of plasmid. Curved grey arrows are intrinsic growth rates of respective cell types. Symbols used for the model are explained in Table 1.

Conjugation and curing is assumed to result in the transformation of one type of cell to another as given in Figure 1. We assume that only one type of plasmid is transferred or cured at a time so that a cell does not go from an uninfected state to a multiple infection state in a single step. Also, a multiple infected cell does not become completely cured of plasmids in single time step.

Let *β_l_* and *β_h_* be the conjugation frequency of the low copy number and high copy number plasmids (plasmid *lc* and *hc* respectively). The dynamics of transfer of one host to another host can be realized from Table 2. Based on Table 2 we can write the differential equations which govern the change in the frequency of different types of cells by adding all the terms that contribute to the change in the cell type. For instance, the differential equation for a plasmid free cell can be given as,
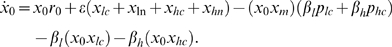
Similarly, the dynamics of change in the proportions of plasmids (*p_lc_*, *p_ln_*, *p_hc_*, *p_hn_*) in the case of multiple infections can be written based on Table 3.

**Table 2:** Dynamics of change in the population of cell types.

Cell type	Cell growth	Addition due to curing of plasmids of other hosts	Decrease in the population due to curing of plasmid	Transfer to other cell type due to receiving plasmid from conjugation	Transferred from other cell type to current cell type due to receiving plasmid from conjugation
*x*_0_	*x*_0_ *r*_0_	*ε* (*x_lc_*+*x_ln_*+*x_hc_*+*x_hn_*)	NA	−*x*_0_ *x_m_*(*β_l_ p_lc_*+*β_h_ p_hc_*)−*β_l_ x*_0_ *x_lc_*−*β_h_ x*_0_ *x_hc_*	NA
*x_lc_*	*x_lc_ r_lc_*	*ε* (*p_lc_ x_m_*)	−*ε x_lc_*	−*x_lc_ x_m_*(1−*p_lc_*)(*β_l_ p_lc_*+*β_h_ p_hc_*)−*β_h_ x_lc_ x_hc_*	*x*_0_ *x_m_ p_lc_* (*β_l_ p_lc_*+*β_h_ p_hc_*)+*β_l_ x*_0_ *x_lc_*
*x_ln_*	*x_ln_ r_ln_*	*ε* (*p_ln_ x_m_*)	−*ε x_ln_*	−*x_ln_ x_m_* (1−*p_ln_*)(*β_l_ p_lc_*+*β_h_ p_hc_*)−*x_ln_* (*β_l_ x_lc_*+*β_h_ x_hc_*)	*x*_0_ *x_m_ p_ln_* (*β_l_ p_lc_*+*β_h_ p_hc_*)
*x_hc_*	*x_hc_ r_hc_*	*ε* (*p_hc_ x_m_*)	−*ε x_hc_*	−*x_hc_ x_m_*(1−*p_hc_*)(*β_l_ p_lc_*+*β_h_ p_hc_*)−*β_l_ x_lc_ x_hc_*	*x*_0_ *x_m_ p_hc_* (*β_l_ p_lc_*+*β_h_ p_hc_*)+*β_h_ x*_0_ *x_hc_*
*x_hn_*	*x_hn_ r_hn_*	*ε* (*p_hn_ x_m_*)	−*ε x_hn_*	−*x_hn_ x_m_*(1−*p_hn_*)(*β_l_ p_lc_*+*β_h_ p_hc_*)−*x_hn_* (*β_l_ x_lc_*+*β_h_ x_hc_*)	*x*_0_ *x_m_ p_hn_* (*β_l_ p_lc_*+*β_h_ p_hc_*)
*x_m_*	*x_m_*(*p_lc_ r_lc_*+*p_ln_ r_ln_*+*p_hc_ r_hc_*+*p_hn_ r_hn_*)	NA	−*ε x_m_*	NA	*x_m_* (*β_l_ x_lc_*+*β_h_ x_hc_*) [*x_lc_* (1−*p_lc_*)+*x_ln_* (1−*p_ln_*)+*x_hc_* (1−*p_hc_*)+*x_hn_* (1−*p_hn_*)]+*x_lc_ x_hc_ β_l_ β_h_*+(*x_ln_*+*x_hn_*)(*β_l_ x_lc_*+*β_h_ x_hc_*)

**Table 3:** Dynamics of change in the proportion of different plasmids in multi-plasmid infected cell.

Plasmid type	Cell growth	Replication of plasmid within host	Addition due to conjugal transfer of the plasmid	Plasmid lost by curing
*p_lc_*	*p_lc_ x_m_*	*p_lc_ x_m_ g_lc_*	*p_lc_ x_m_* (*x_ln_* + *x_hc_* + *x_hn_*)(*β_l_ p_lc_* + *β_h_ p_hc_*) + *β_l_ p_lc_* (*x_ln_* + *x_hc_* + *x_hn_*) + *β_h_ x_lc_ x_hc_*	−*ε x_m_ p_lc_*
*p_ln_*	*p_ln_ x_m_*	*p_ln_ x_m_ g_ln_*	*p_ln_ x_m_* (*x_lc_* + *x_hc_* + *x_hn_*)(*β_l_ p_lc_* + *β_h_ p_hc_*) + *x_ln_* (*x_lc_* + *x_hc_* + *x_m_* (1−*p_ln_*))(*β_l_ p_lc_* + *β_h_ p_hc_*)	−*ε x_m_ p_ln_*
*p_hc_*	*p_hc_ x_m_*	*p_hc_ x_m_ g_hc_*	*p_hc_ x_m_* (*x_lc_* + *x_ln_* + *x_hn_*)(*β_l_ p_lc_* + *β_h_ p_hc_*) + *β_h_ p_hc_* (*x_lc_* + *x_ln_* + *x_hn_*) + *β_l_ x_lc_ x_hc_*	−*ε x_m_ p_hc_*
*p_hn_*	*p_hn_ x_m_*	*p_hn_ x_m_ g_hn_*	*p_hn_ x_m_* (*x_lc_* + *x_ln_* + *x_hc_*)(*β_l_ p_lc_* + *β_h_ p_hc_*) + *x_hn_* (*x_lc_* + *x_hc_* + *x_m_* (1−*p_hn_*))(*β_l_ p_lc_* + *β_h_ p_hc_*)	−*ε x_m_ p_hn_*

### Simulations

Analytical solutions were possible when only one type of plasmid or two types of host cells were present. Considering all plasmid types and cell types was difficult to study analytically owing to the inherent complexity of the model. Therefore we analyzed the model numerically. All simulations began with a plasmid free cell population to which all plasmid types were invaders. We assumed that any of the plasmid types can arise by mutation and a low frequency of mutation selection balance was maintained for all the types so that they had a fair chance of back invasion at any point in time. The total population of host cells as well as that of plasmids in *x_m_* was normalized to unity at the end of each time unit.

To study the effect of copy number increase with small increments, we adopted the following approach. If an *lc* plasmid was successfully invaded by an *hc* mutant, the parameters of the *hc* invader were made the parameters of *lc* in the following round of simulations and a new *hc* with slightly higher copy number was introduced. At each turn of the simulation cycle back invasion by the initial low copy number wild type was also attempted. A similar approach was adopted to study the effects of other parameters such as conjugation efficiency.

## Results

When only the wild type (*lc*) plasmid was present, equilibrium was established between the plasmid free and plasmid bearing cells owing to a balance between the greater inter-host fitness of plasmid free population, plasmid transfer by conjugation and spontaneous plasmid curing. The differential equations governing the change in population of plasmid free cells and *lc* plasmid containing cells can be given as
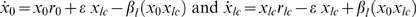
At equilibrium,
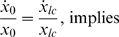


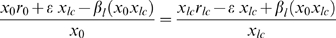
It can be seen that as *x_lc_*+*x_0_* = 1, the stable ratio at equilibrium would be
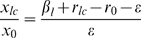
Numerical simulations matched the analytical solution.

**Figure 2:**
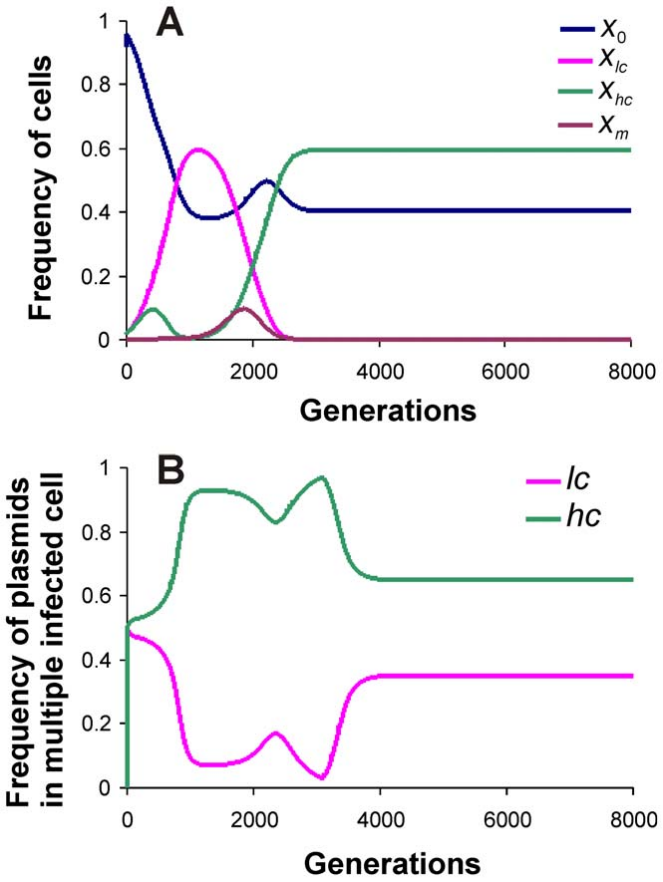
Invasion by a mutant plasmid with higher fitness (*hc* plasmid). (A) Populations of host cells and (B) proportions of plasmids in host cells with multiple infection. Parameter values for the figure are *μ* = 0.2, *λ* = 0.02, *φ* = 0.004, *ε* = 0.01, *β_l_* = 0.05, *β_h_* = 0.05, *δ* = 0.5.

**Figure 3:**
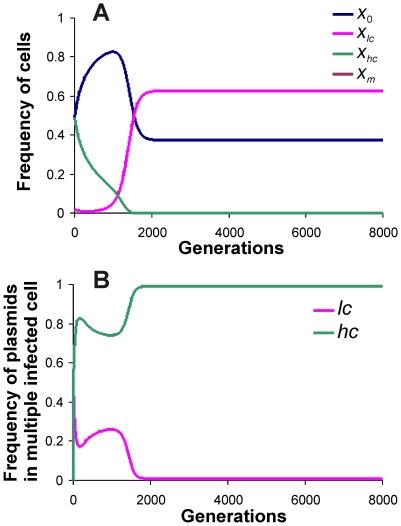
Mutant plasmid with exorbitantly high copy number is invaded back by wild type plasmid. (A) Populations of host cells and (B) proportions of plasmids in host cells with multiple infection. *δ* = 5 all other parameters as in Fig 2.

When a mutant with a higher copy number (*hc* plasmid) was introduced in the presence of wild type (*lc*) plasmid, provided the difference in copy number was not too large, the wild type was driven to extinction or very low frequencies by the invader (Figures 2A and 2B). This was obvious as the mutant had more intra-host fitness than the wild type plasmid. The mutant with a higher copy number was vulnerable to invasion by another mutant that had higher copy number, which in turn was vulnerable to invasion by another mutant with still higher copy number and so on. Thus, mutants with slightly higher fitness invaded the ones with lower fitness. This process could increase the copy number to a very large value especially when the host cost of plasmid fitness (i.e. cost of carrying additional plasmid copies) was small. However, at some stage the wild type plasmid with low copy number could back invade the high copy number population (Figures 3A and 3B). This was owing to the large metabolic burden on the host cell carrying the high copy number plasmid. The back invasion was possible only when the copy number of the invading variant was substantially smaller. The back invading low copy plasmid was eventually susceptible to invasion by slightly high copy number variant. Therefore the system did not have a unique stable equilibrium.

**Figure 4:**
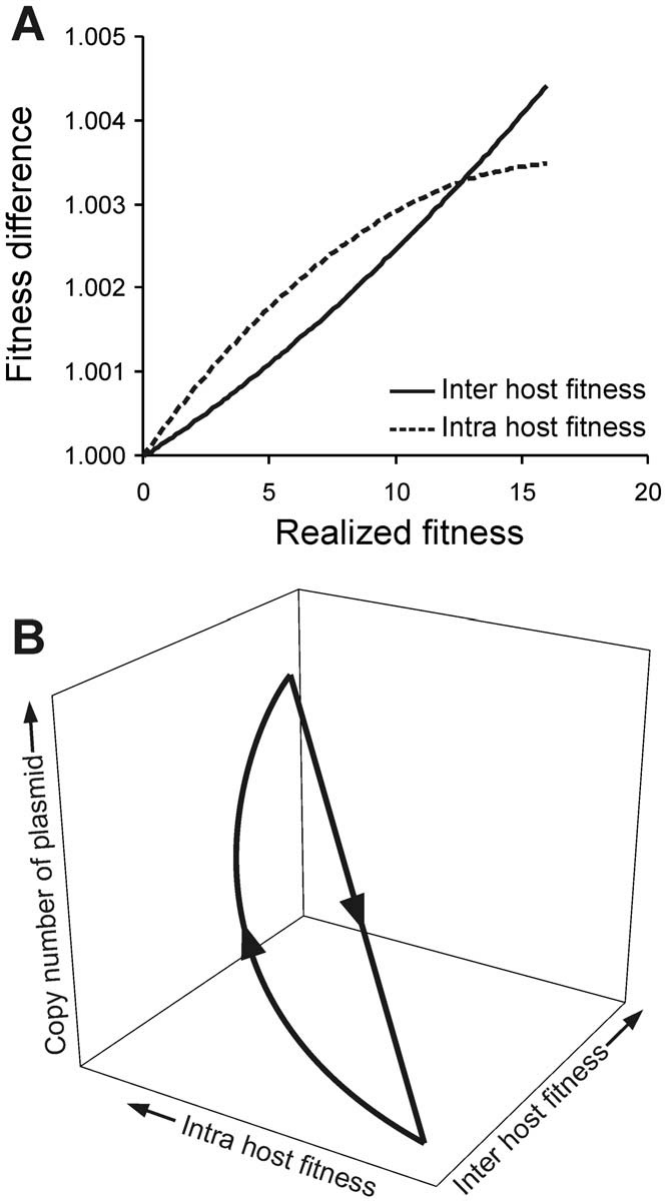
A conceptual diagram based on generalization from many numerical results, depicting the effect of intra and inter-host fitness on realized fitness. (A) The realized fitness is a saturating function of intra-host fitness and an exponential function of inter-host fitness. As a result of the different nature of the two curves, RPS like dynamics results which is depicted schematically in (B). Arrows indicate the direction of evolutionary change. Mutants with slightly higher copy numbers can invade lower copy number plasmids owing to higher intra-host fitness. As a result the mean copy number increases, however the effect of intra-host fitness is saturating and therefore at a certain stage the reducing positive increment in realized fitness is over-compensated by increasing negative contribution of inter-host fitness difference. At this stage a low copy number plasmid can back invade resulting into a cyclic evolutionary dynamics.

The major reason for this dynamics was the difference in the effects of inter and intra-host fitness. In order to differentially study and compare the effects of the two we defined realized fitness as the net increment in the total (including all host cells) plasmid proportion over time *t* (*t* = 100 in the simulations). A common problem with multi level selection is that the different levels of selection work with different net effects on the realized fitness. We took the approach of studying the nature of the relationship between fitness levels with realized fitness taking one level at a time. To analyze the effects of intra-host fitness on realized fitness, inter-host fitness difference was set to zero by making *φ* = 0 and the increment in the net population of plasmid over time *t* was monitored at different values of *δ*. Inversely to study the realized inter-host fitness in isolation, *δ* was set to zero and the increment in the net population of the plasmid over time *t* was monitored at increasing inter-host fitness. It was observed that with increasing *δ* the realized fitness increased in a saturation curve. On the other hand, the realized fitness increased exponentially with increasing inter-host fitness difference as shown schematically in Figure 4A and B. The saturating nature of the realized fitness curve with increasing intra-host fitness was owing to the inherent limitation on growth within a host cell and a constant and limiting rate of transfer across cells. Although a plasmid with a very high copy number could replace the wild type very rapidly within a host cell clone, its spread to other cells was not equally rapid being limited by the rate of conjugal transfer which was assumed to be constant. As a result of the two different natures of the curves, with a small increment in copy number the intra-host fitness advantage dominated over the inter-host fitness but with a larger difference, the inter-host fitness increased more sharply to overcompensate for the saturating effects of intra-host fitness and resulted in a net loss to the high copy number plasmid.

The resulting dynamics were similar to Rock-Paper-Scissors (RPS) game [26]–[28] where plasmids with low copy number were successively invaded by plasmids with higher copy number due to the increase in the intra-host fitness till a point where the intrinsic growth rate of the host was greatly reduced, due to increased metabolic burden, and owing to the inter-host fitness difference the plasmids with lower copy number could back invade the population and the cycle started again (Figure 4B). It is well known that RPS game leads to oscillating or stable coexistence of all the three strategies [26]–[28]. Therefore we expect that low intermediate and high copy number plasmids could coexist in a host species in a dynamic equilibrium. The only difference between the classical RPS game and the plasmid dynamics is that the mean copy numbers of plasmids can vary on a single continuous scale and therefore have a number of intermediates while classically rock-paper-scissor are three qualitatively distinct and discrete strategies.

Introduction of the second type of cheating in the form of *ln* and *hn* plasmids changed the picture considerably. Plasmids *ln* and *hn* were assumed not to carry the *tra* gene complex required for conjugation but in the presence of *lc* or *hc* plasmids they could get transferred to other hosts through conjugal hitchhiking. The absence of *tra* gene complex was assumed to reduce cost and thereby increase the fitness of the plasmid as well as the host carrying it. This led to an increase in efficient vertical transfer of the plasmid but the horizontal transfer was restricted from host cells co-infected with *lc* and/or *hc* and *ln* and/or *hn*. In pair wise interactions *ln* plasmid could invade (but not replace) *lc* plasmid but not the *hc* plasmid. The invasion was restricted and led to a stable coexistence of the wild type and cheater. Since the cheater depended upon the wild type for conjugal transfer, it could not drive the wild type to extinction. Plasmid *hn* could invade (but not replace) *lc* as well as *hc* plasmids and also outcompeted *ln* invariably.

**Figure 5:**
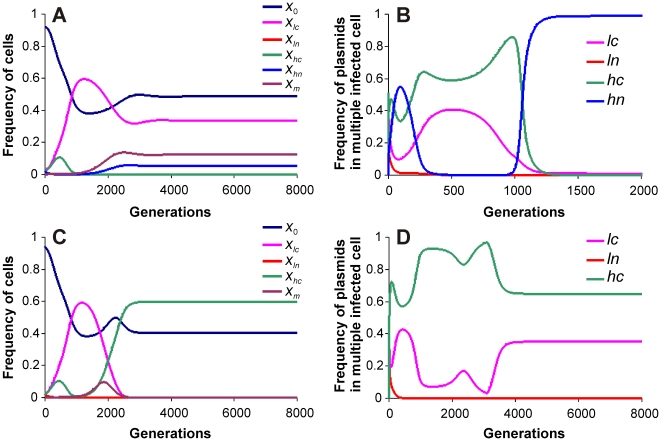
The interaction of the two types of cheaters. Successful invasion of *hc* plasmid by *hn* plasmid in cells with multiple infection brings the wild type back (A, B). Parameter values for the figure are as per Fig. 2. In the absence of *hn* plasmid, for the same parameters, *hc* plasmid dominates the population (C and D).

When all the four types were simultaneously included in the simulations there were interesting consequences of the interaction between the two types of cheaters. Whenever the *hn* plasmid successfully established in the population it reduced the fitness advantage of *hc*. Since *hn* shared the benefit of higher copy number with *hc* and also had a lower cost, it had a higher intra-host fitness then *hc*. In a typical result (Figure 5A and 5B) *hc* rapidly increased in frequency but was soon followed by *hn* which largely replaced *hc* in co-infected host. The reduction in the frequency of *hc* was followed by back invasion by *lc*. Since *hn* had a limited capacity to invade *lc*, both *lc* and *hn* coexisted in a stable steady state where the pure plasmid host population was dominated by *lc* and co-infected host population was dominated by *hn*. Keeping other parameters the same omission of *hn* resulted in invasion by *hc* and near extinction of *lc* (Figures 5C and 5D).

**Figure 6:**
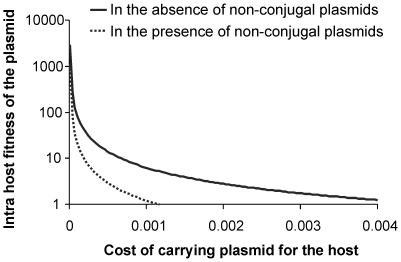
The maximum intra-host plasmid fitness as a function of plasmid cost. The lines show the limit of intra-host fitness above which the wild type can back invade. In presence of the non-conjugal cheaters the limit is substantially reduced. All other parameters as in Fig 2.

Across a wide range of costs of the plasmid (Figure 6) the non-conjugal plasmids were observed to restrict the width of copy numbers over which the RPS situation worked. The low copy number wild type was able to back invade the higher copy mutants at a much smaller copy number when non-conjugal plasmids were present. The two modes of cheating thus had antagonistic effects resulting into effective arrest of escalation. The RPS dynamics itself was robust to changes in other parameters. Throughout the range of parameters that did not result into extinction of all types of plasmids, low copy number plasmids could exist either stably or in RPS dynamics. The RPS limit lines (Figure 6) were unaffected by changes in *ε* as long as it was independent of the copy number. Both *μ* and *λ* pushed down the limit line marginally in the absence of cheaters. However, in presence of non-conjugal cheaters *μ* exerted a strong effect in lowering the RPS limit whereas *λ* had a marginal effect. In other words increase in *μ* helped the stability of low copy number plasmids in presence of non-conjugal cheaters.

Unlike the fitness effect of copy number, the conjugation efficiencies *β_l_* and *β_h_* had a linear effect on fitness. Plasmid with higher conjugation efficiency was selected over one with lower efficiency when other parameters were equal. Therefore although conjugal and non-conjugal plasmids coexisted, coexistence of different conjugation efficiencies was not possible. Since selection for conjugation efficiencies was linear, there was a unidirectional selection for highest conjugation efficiency. We must assume then that the rate of conjugation is limited by physical factors and availability of recipient cells rather than the efficiency of conjugation machinery.

Reducing the probability of conjugal transfer to any cell with pre-existing plasmid reduced the frequency of co-infection. This could effectively make intra-host selection weaker and as a result, low copy number plasmids increased substantially in frequency. Prevention of co-infection can therefore enhance the stability of low copy numbers. However, the model showed that preventing co-infection was not necessary for the stability of low copy numbers.

## Discussion

The model shows that low copy number plasmids could be maintained in spite of a threat by copy-up mutants owing to a continuous RPS like relationship in mutants with low, intermediate and high copy numbers. Although multi-level selection in plasmids [16] and other host parasite interactions [29], [30] has been examined before, co-existence mediated by an RPS like situation was not predicted so far. Paulsson [16] realized that a selfish plasmid was at a net advantage “…as long as it is not too selfish” but failed to extend this logic to visualize the RPS like consequence of it. The RPS dynamics results from the two types of non-linear effects of intra and inter-host fitness. Inter-host fitness working in an exponentially growing host population has an exponential effect on the realized fitness. On the other hand, intra-host fitness has an inherent upper limit since they apply only to the currently infected host. The saturation is also owing to the rate limiting nature of conjugation. Interaction of these two types of curves depicted in Figure 4 is fundamental to the central result of the model.

The assumption of rate limiting nature of conjugation is not arbitrary. The efficiency of conjugation does not bear a saturating relationship with realized fitness and therefore both low and high copy number plasmids should evolve conjugation machinery with high efficiency. The conjugation rate then should be limited by physical factors and the probability of finding recipient cells. Therefore the conjugation efficiency can be assumed to be constant independent of the intra-host fitness.

The results of this model may be generalized to any situation of conflict between intra-group and inter-group selection. It is likely to work similarly and give rise to RPS mediated coexistence if only one basic requirement is met with, that is the intra-group fitness advantage needs to be a saturating function. It is natural for the inter-group fitness advantage to be exponential since most biological populations are assumed to replicate giving rise to exponential kinetics. Generalizing the model is likely to give us more insights into the dynamics of group selection and conflicts between levels of selection.

If cells with preexisting plasmids refuse to accept a conjugal transfer the frequency of co-infection will be reduced making inter-host fitness stronger. This strategy however is not fool proof since co-infections can also arise by mutations within cells. Secondly the non-conjugal plasmids should evolve to welcome co-infection by accepting conjugal transfer since they can increase their fitness in co-infected cells. Therefore prevention of co-infection by incompatibility or rendering conjugation immunity to the host cell is unlikely to be a stable and effective strategy by itself. Our model shows, on the other hand that low copy numbers can be effectively maintained even without prevention of co-infection. Therefore this mechanism appears neither necessary nor sufficient but nevertheless helpful for the stability of low copy numbers. An important testable prediction of the model is that low copy number conjugal plasmids should confer immunity to the host against conjugal invasion whereas high copy number non-conjugal plasmids should welcome conjugal invasion by other plasmids. Another testable prediction of the model is that plasmids with different copy numbers would be present in any given host species within a single population or metapopulations. Metapopulation dynamics is likely to affect coexistence further which has not been examined as yet but which is beyond the scope of this paper. Nevertheless the model predicts variability in plasmids in a host population and variations in copy number and associated cost have indeed been shown by plasmids over different host strains [3] and over time [4]–[6]. It would also be possible to study the long term dynamics of plasmid varieties in a sufficiently large host population using a continuous culture system and using carefully selected plasmid types in pairs and in complex combinations.

The further twist to the story added by the second type of cheating is not hypothetical alone. It is well known that non-conjugal plasmids are common and they can undergo conjugal transfer in presence of conjugal plasmids [9], [31]–[33]. Two mechanisms can be employed by the non-conjugal plasmids for hitch-hacking. First method is called mobilization, a process by which plasmids achieve transfer by ‘borrowing’ the gene products of a conjugal plasmid, as in the case of ColE1 and RSF1010 plasmids of *Escherichia coli*. The second method is called conduction, which involves a physical association with conjugal plasmid forming co-integrate that may resolve into two plasmids in the recipient. Our model predicts co-existence of conjugal and non-conjugal plasmids along with low and high copy number plasmids and further that non-conjugal plasmids with very low copy numbers are unlikely to be stable since *ln* plasmids were always invaded by *hn*. Therefore all non-conjugal plasmids should have high copy numbers. This can be taken as another testable prediction of the model. The interaction between the two types of cheating is important for the maintenance of plasmid diversity along both the axes.

While evolution would have shaped both molecular and sociobiological processes, much research has focused on the molecular mechanisms of plasmid replication dynamics and the sociobiological interactions have largely been ignored. We tried to develop some insights in the possible sociobiological factors in plasmid dynamics. It would be interesting to further elucidate the interplay between molecular and sociobiological mechanisms in plasmid dynamics.

## References

[1] Freter R, Freter RR, Brickner H (1983). Experimental and mathematical models of *Escherichia coli* plasmid transfer *in vitro* and *in vivo*.. Infection Immunity.

[2] Gelder LD, Ponciano JM, Abdo Z, Joyce P, Forney LJ, Top EM (2004). Combining mathematical models and statistical methods to understand and predict dynamics of antibiotic-sensitive mutants in a population of resistant bacteria during experimental evolution.. Genetics.

[3] Gelder LD, Ponciano JM, Joyce P, Top EM (2007). Stability of a promiscuous plasmid in different hosts: no guarantee for a long-term relationship.. Microbiol.

[4] Lenski RE, Simpson SC, Nguyen TT (1994). Genetic analysis of a plasmid-encoded, host genotype-specific enhancement of bacterial fitness.. J Bacteriol.

[5] Dahlberg C, Chao L (2003). Amelioration of the cost of conjugative plasmid carriage in *Eschericha coli* K12.. Genetics.

[6] Dionisio F, Conceicao IC, Marques ACR, Fernandes L, Gordo I (2005). The evolution of a conjugative plasmid and its ability to increase bacterial fitness.. Biol Lett.

[7] Smith MA, Bidochka MJ (1998). Bacterial fitness and plasmid loss: importance of culture conditions and plasmid size.. Canadian J Microbiol.

[8] Corchero JL, Villaverde A (1998). Plasmid maintenance in *Escherichia coli* recombinant cultures is dramatically, steadily and specifically influenced by features of the encoded proteins.. Biotechnol Bioengineering.

[9] Summers DK (1996). The biology of plasmids.

[10] Williams PA (2004). Catabolic plasmids: fast-track bacterial evolution to combat pollution.. Microbiol Today.

[11] Bergstrom CT, Lipsitch M, Levin BR (2000). Natural selection, infectious transfer and the existence conditions for bacterial plasmids.. Genetics.

[12] Smith J (2001). The social evolution of bacterial pathogenesis.. Proc Royal Soc B.

[13] Yarmolinsky MB (2000). A pot-pourri of plasmid paradoxes: effects of a second copy.. Mol Microbiol.

[14] Zielenkiewicz U, Cegłowski P (2001). Mechanisms of plasmid stable maintenance with special focus on plasmid addiction systems.. Acta Biochimica Polonica.

[15] Lili LN, Britton NF, Feil EJ (2007). The persistence of parasitic plasmids.. Genetics.

[16] Paulsson J (2002). Multilevel selection on plasmid replication.. Genetics.

[17] Velmurugan S, Mehta S, Uzri D, Jayaram M (2003). Stable propagation of ‘selfish’ genetic elements.. J Biosci.

[18] Abhyankar MM, Reddy JM, Sharma R, Bullesbach E, Bastia D (2004). Biochemical investigation of control of replication initiation of plasmid R6K.. J Biol Chem.

[19] del Solar G, Giraldo R, Ruiz-Echevarría MJ, Espinosa M, Díaz-Orejas R (1998). Replication and control of circular bacterial plasmids.. Microbiol Mol Biol Rev.

[20] Projan SJ, Monod M, Narayanan CS, Dubnau D (1987). Replication properties of pIM13, a naturally occurring plasmid found in *Bacillus subtilis*, and of its close relative pE5, a plasmid native to *Staphylococcus aureus*.. J Bacteriol.

[21] Acebo P, Alda MT, Espinosa M, del Solar G (1996). Isolation and characterization of pLS 1 plasmid mutants with increased copy numbers.. FEMS Microbiol Lett.

[22] Chattoraj D, Cordes K, Abeles A (1984). Plasmid P1 replication: Negative control by repeated DNA sequences.. Proc Natl Acad Sci USA.

[23] Das N, Valjavec-Gratian M, Basuray AN, Fekete RA, Papp PP (2005). Multiple homeostatic mechanisms in the control of P1 plasmid replication.. Proc Natl Acad Sci USA.

[24] Paulsson J, Ehrenberg M (2001). Noise in a minimal regulatory network: plasmid copy number control.. Quart Rev Biophysics.

[25] Novick RP (1987). Plasmid incompatibility.. Microbiol Rev.

[26] Sinervo B, Lively CM (1996). The rock-paper-scissors game and the evolution of alternative male strategies.. Nature.

[27] Hauert C, De Monte D, Hofbauer J, Sigmund K (2002). Volunteering as red queen mechanism for cooperation in public goods game.. Science.

[28] Kerr B, Riley MA, Feldman MW, Bohannan BJM (2002). Local dispersal promotes biodiversity in a real-life game of rock–paper–scissors.. Nature.

[29] Nowak MA, May RM (1994). Superinfection and the evolution of parasite virulence.. Proc Royal Soc B.

[30] Mideo N, Alizon S Day T (2008). Linking within- and between-host dynamics in the evolutionary epidemiology of infectious diseases.. Trends Ecol Evol.

[31] Andrup L, Smidt L, Andersen K, Boe L (1998). Kinetics of conjugative transfer: a study of the plasmid pxo16 from *Bacillus thuringiensis* subsp *israelensis*.. Plasmid.

[32] Flett F, Humphrey GO, Saunders JR (1981). Intraspecific and intergeneric mobilization of non-conjugative resistance plasmids by a 24.5 megadalton conjugative plasmid of *Neisseria gonorrhoeae*.. J Gen Microbiol.

[33] Udo EE, Jacob LE, Mokadas EM (1997). Conjugative transfer of high-level mupirocin resistance from *Staphylococcus haemolyticus* to other staphylococci.. Antimicrobial Agents Chemotherapy.

